# 45 km ROTDR with 0.5 m/0.11 °C via complex-domain square-wave width-chirp pulse compression

**DOI:** 10.1038/s41377-026-02245-1

**Published:** 2026-03-16

**Authors:** Bowen Fan, Jian Li, Xinyue Zhang, Lulei Li, Rilong Wang, Jianzhong Zhang, Mingjiang Zhang

**Affiliations:** 1 College of Physics and Optoelectronics, Taiyuan University of Technology, Taiyuan, China; 2 Key Laboratory of Advanced Transducers and Intelligent Control System, Ministry of Education, Taiyuan University of Technology, Taiyuan, China; 3 Shanxi Key Laboratory of Precision Measurement Physics, Taiyuan University of Technology, Taiyuan, China

**Keywords:** Fibre optics and optical communications, Imaging and sensing

## Abstract

Raman optical time-domain reflectometry (ROTDR) inherently balances sensing range, spatial resolution, and temperature accuracy through the pulse duration dictated by the OTDR position principle. However, optimizing one metric conventionally degrades the others, forming a theoretical trade-off. This work introduces complex-domain square-wave width-chirp pulse compression to break that physical limitation. The steep edges and rich high-order harmonics of complex-domain square-wave width-chirp pulse undergo matched filtering, producing a compressed δ-pulse whose full width at half maximum, rather than the original pulse duration, now governs sensing spatial resolution. Complex-domain matched filtering, implemented via a conjugate time-reversal filter, achieves a 15.09 dB gain in signal-to-noise ratio, while the complex-domain envelope extraction method isolates and removes Raman phase noise. The proposed scheme simultaneously achieves 45 km sensing distance, 0.5 m spatial resolution, and 0.11 °C temperature accuracy, demonstrating complete decoupling of these metrics from the pulse duration. The proposed framework offers a new paradigm for long-range, high-precision distributed temperature sensing and is extensible to Brillouin and Rayleigh scattering systems.

## Abstract (web summary)

This work proposes a complex-domain square-wave width-chirp pulse compression scheme, achieving simultaneous improvement of spatial resolution, sensing distance, and temperature accuracy in the field of Raman distributed fiber optic sensing.

## Graphical abstract

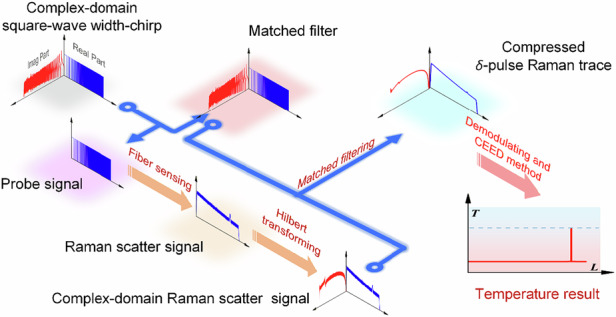

## Introduction

In the information era, smart sensors have become essential tools for acquiring data from both natural and human-made environments^1–9^. Among them, distributed optical fiber sensors^10–15^ offer unparalleled advantages—such as long-range coverage, intrinsic safety, immunity to electromagnetic interference, and cost-effectiveness—making them indispensable for monitoring physical parameters, including temperature, strain, and vibration. Raman optical time-domain reflectometry (ROTDR), which leverages spontaneous Raman scattering, is particularly well-suited for temperature sensing due to its exclusive temperature sensitivity^16–20^. It has been widely deployed in applications ranging from infrastructure health diagnostics and fire warning systems to oil and gas pipeline monitoring.

Despite its advantages, the performance of ROTDR systems remains fundamentally constrained by the trade-off among spatial resolution, sensing range, and temperature accuracy^21^. The spatial resolution is directly limited by the temporal width of the probing pulse. Within a single pulse duration, backscattered Raman signals from different spatial segments overlap, resulting in a spatially averaged temperature signal that obscures fine-scale variations^22^.

The most direct method to improve spatial resolution is to compress the pulse duration. Previous studies have demonstrated that centimeter-level resolution is achievable at meter-scale ranges using ultra-narrow pulses or superconducting detectors^23–26^. Pulse duration compression results in a significant reduction of the pulse energy launched into the fiber, leading to a sharp decline in signal-to-noise ratio (*SNR*)—a critical factor that determines both the maximum sensing range and the accuracy of temperature extraction^27^. Although increasing the peak power of the pulse could compensate for the energy loss, this approach is fundamentally limited by fiber nonlinearities (e.g., SBS^28,29^ or SRS^30,31^) and the damage thresholds of lasers and detectors. Consequently, ROTDR systems face a core performance dilemma: enhancing spatial resolution requires narrower pulses, but maintaining sufficient *SNR*—and hence long range and high precision—requires higher pulse energy.

To address this intrinsic limitation, algorithmic solutions have been proposed that aim to reconstruct high-resolution temperature information from weak Raman signals. These include differential reconstruction techniques, which extract intra-pulse detail via self-pulse^32^ or dual-pulse comparison^33^, and deconvolution approaches employing neural networks^34–37^, which attempt to reverse the convolution effect of finite pulse duration. While these methods offer a promising route to improve spatial resolution without physically shortening the pulse, they are inherently sensitive to the *SNR* of the raw backscattered signal^38^. For example, differential methods amplify random noise while extracting fine-scale features^33^, and deep-learning models may introduce artifacts, overfit noise, or exhibit unstable outputs when operating on low-*SNR* inputs^36^. These challenges restrict their practical utility in long-distance sensing scenarios, where signal attenuation inherently reduces the *SNR*. As a result, although algorithmic optimization enables improved resolution over several kilometers, it does not fundamentally overcome the core trade-offs dictated by *SNR* constraints.

High-entropy light source sensing methods have also been explored to bypass the spatial resolution limitation imposed by pulse duration^37–41^^.^ These methods utilize light sources with broad spectra and low coherence—such as chaotic lasers^38–40^ and amplified spontaneous emission sources^41,42^—to generate temporally random waveforms. By computing the cross-correlation between the backscattered signal and a reference copy, the position of temperature events can be resolved with spatial resolution determined by the correlation peak width, rather than the duration of the input pulse. In proof-of-concept experiments, spatial resolution as fine as 0.3 m has been demonstrated over a 10 km sensing length, with temperature accuracy of approximately 2.0 °C. These approaches offer resolution enhancement without pulse compression and are especially promising for mid-range applications. However, their extension to longer distances is hindered by signal degradation. In particular, the inherently weak Raman signal undergoes distortion and broadening due to chromatic dispersion and attenuation during fiber propagation, resulting in smeared or lost correlation features that compromise performance at long ranges.

To extend the sensing distance of ROTDR systems beyond existing limits, other solutions have been proposed involving specialty fiber designs^43–45^, pulse coding strategies^46,47^, and wavelength shifting of the pump source^48^. Among them, specialty fibers such as graded-index few-mode fibers enhance the efficiency of Raman scattering and reduce modal dispersion, allowing for a 1.13 m resolution over 25.0 km^44^. Low water-peak fibers offer reduced loss in the 1550 nm transmission window and have achieved 1.0 m resolution over 24.0 km^45^. Pulse coding techniques such as quasi-periodic low-duty-ratio coding and optimized aperiodic sequences increase effective pulse energy under peak power limits, resulting in 1.0 m resolution at 39.0 km sensing lengths^47^. Raman wavelength-shifting schemes relocate the excitation wavelength to 1630 nm, such that the anti-Stokes signal falls within the fiber’s lowest-loss band at 1550 nm. This strategy has achieved sensing distances up to 85.0 km with a spatial resolution of 800 m and temperature accuracy of 8 °C^48^.

While these developments mark significant progress, they also highlight the persistent trade-offs in ROTDR performance. Pulse coding methods extend the range while maintaining meter-scale resolution, but often at the cost of temperature accuracy(3.9 °C at 39 km^47^) and increased system complexity. Wavelength-shifted systems demonstrate exceptional range but suffer from coarse spatial resolution and poor temperature accuracy^48^ (800 m and 8 °C). As such, these solutions only partially alleviate the underlying trade-offs among resolution, range, and accuracy, without fundamentally resolving the ROTDR’s system trilemma.

Zou et al. proposed an optical pulse compression reflectometry (OPCR) scheme^49,50^ and applied it to a Rayleigh-scattering OTDR sensing system. The key advantage of this scheme is that the spatial resolution is determined by the frequency-sweeping bandwidth of the chirped probing signal, rather than by the initial pulse duration as in a traditional scheme. As a result, OPCR achieves a substantially improved trade-off between spatial resolution and sensing range. Rayleigh-scattering sensing systems based on OPCR can extend the effective sensing distance to several times the laser coherence length; experiments have demonstrated a spatial resolution of 0.47 m over a 5 km fiber link^49^. By combining double-sideband modulation technology, OPCR achieves an excellent sensing performance with a spatial resolution of 10 cm over a sensing distance of 58 km^50^.

However, in ROTDR systems, the spontaneous Raman backscattered signal is extremely weak and exhibits poor coherence. These characteristics can severely obscure the instantaneous-frequency signature of the chirped probing signal, thereby preventing OPCR from being directly applicable to ROTDR. Therefore, a fundamental re-architecture of the technique is required to enable practical deployment in ROTDR systems and to ensure that temperature information can be demodulated accurately and reliably.

Here, we propose a complex-domain square-wave width-chirp pulse compression (CSWPC) scheme that simultaneously enhances spatial resolution, sensing distance, and temperature accuracy in ROTDR systems. In CSWPC, the chirp-induced instantaneous-frequency evolution is encoded as a monotonic width modulation of square-wave sub-pulses, and the received real-valued Raman backscattered signal is converted into a complex analytic signal via the Hilbert transform, enabling synchronous extraction of the amplitude envelope and instantaneous-frequency information. Leveraging the sharp temporal transitions and rich high-order harmonics of the complex-domain square-wave width-chirped probing pulse, the proposed method generates a compressed, δ-like response after pulse compression, thereby overcoming the conventional spatial-resolution limit imposed by pulse duration. A complex-domain matched filter maximizes the output *SNR*, while complex-domain envelope extraction denoising (CEED) further improves signal fidelity and suppresses phase-noise-induced fluctuations in temperature demodulation. Experimentally, we demonstrate 0.5 m spatial resolution and 0.11 °C accuracy over a 45 km fiber, representing the first reported sub-meter, high-accuracy, ultra-long-range distributed Raman sensing system. This scheme also provides a generalizable framework for performance enhancement in scattering-based distributed fiber sensing technologies.

## Results

### Principle and demodulation scheme

**Fig. 1:**
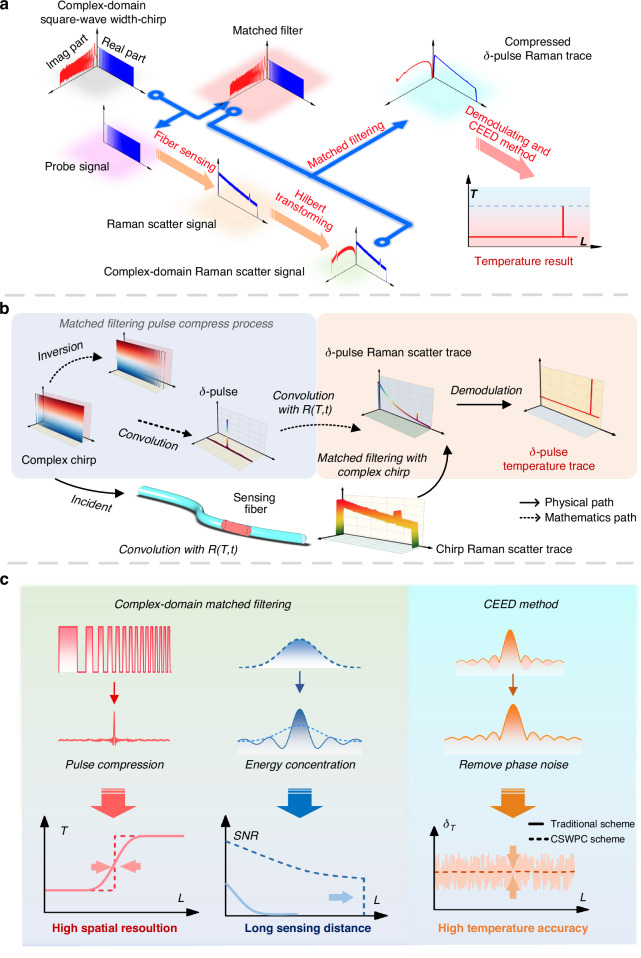
CSWPC sensing scheme and physics temperature demodulation principle. **a** CSWPC scheme. **b** Physics principle of obtaining the δ-pulse Raman scattering trace and the corresponding temperature distribution through complex-domain match-filtering. **c** Principle of simultaneous enhancement in spatial resolution, sensing distance, and temperature accuracy by the CSWPC scheme. *R*(*T,t*) Raman characteristic equations. CEED complex-domain envelope extraction denoising

This work establishes a theoretical model named CSWPC that achieves pulse compression and energy focusing of the probe signal through complex-domain matched filtering, thereby enabling simultaneous improvement in spatial resolution and *SNR* in ROTDR systems, as illustrated in Fig. 1. In Fig. 1a, a complex-domain square-wave width-chirp pulse signal is employed both as the probe and the matched filter. After propagating through the sensing fiber, the probe excites spontaneous Raman backscattering, which is subsequently processed via a Hilbert orthogonal transform to obtain the complex-domain Raman signal. This signal is then processed using complex-domain matched filtering, yielding a compressed Raman response that is equivalent to a δ-pulse in the time domain. To achieve quantitative mapping from the signal amplitude to physical temperature, this paper introduces a chirp matching coefficient *A*, establishing a complete complex-domain amplitude–distributed temperature demodulation model that, with CEED, the temperature *T* at fiber location *L* can be retrieved using Eq. (1).

1 $$T=\frac{{\mathrm{h}}\Delta \nu }{{\rm{k}}_{\rm{B}}\,{\mathrm{ln}}\left[\frac{{\mathrm{abs}}({\mathop{M}\limits^{\frown {}}}_{0}(L))}{{\mathrm{abs}}(\mathop{M}\limits^{\frown {}}(L))}\cdot \frac{{\mathrm{exp}}({\rm{h}}\varDelta \nu /{\rm{k}}_{\rm{B}}{T}_{0})-1}{A}+1\right]}$$

Where h is the Planck constant, Δ*ν* is the Raman frequency shift, k_B_ is the Boltzmann constant, and abs(·) is the extraction amplitude envelopes in the complex domain (The CEED method). ${\hat{M}}_{0}$ and $\hat{M}$ are the compressed δ-pulse Raman scattering traces at room temperature *T*_0_ and during measurement, respectively. *T*_0_ is room temperature. *A* is the chirp matching coefficient.

In response to the characteristic that traditional spontaneous Raman scattering is insensitive to the frequency and phase of the light source, this paper introduces the Hilbert transform to convert the real-valued optical intensity signal into a complex analytic signal. On this basis, complex-domain matched filtering is employed to achieve signal amplitude and phase decoupling, thereby simultaneously obtaining the amplitude envelope and instantaneous frequency of the Raman scattering signal without relying on a coherent detection architecture.

The principle of obtaining the compressed δ-equivalent Raman scattering trace and the corresponding temperature distribution trace through matched filtering is illustrated in Fig. 1b. In mathematics, the matched filtering process for the complex-domain square-wave width-chirp pulse is equivalent to time-reversing the signal and convolving it with itself, resulting in a δ-pulse with an extremely narrow full width at half maximum (*FWHM*). In physics, the spontaneous Raman scattering excited by this δ-pulse in the fiber can be interpreted as a time-domain convolution between the δ-pulse and the spontaneous Raman response function. By the commutative property of convolution, this process is equivalent to matched filtering between the Raman scattering response of the complex-domain square-wave width-chirp pulse signal and the complex-domain square-wave width-chirp pulse signal itself. Through this matched filtering scheme, an equivalent δ-pulse Raman scattering curve is obtained, from which the temperature profile is demodulated. The spatial resolution of the temperature sensing is thus determined by the *FWHM* of the δ-pulse.

Figure 1c illustrates the principle of simultaneous enhancement in spatial resolution, sensing distance, and temperature accuracy enabled by the CSWPC scheme.

When employing the CSWPC scheme, the *FWHM* of the resulting δ-pulse can be approximated by Eq. (2), indicating a spatial resolution that is inversely proportional to the pulse duration. Meanwhile, the system *SNR*, which governs sensing range and temperature accuracy, is expressed in Eq. (3). Where c is the speed of light in a vacuum, and n is the refractive index of the fiber, *β* is the chirp rate, *τ* is the pulse duration, and *E*_pulse_ is the pulse energy. This suggests that increasing the pulse duration enhances the coupled optical energy, thereby improving the *SNR*.

2 $${R}_{s}=\frac{{\rm{c}}}{2{\rm{n}}}\mathrm{FWHM}\approx \frac{{\rm{c}}}{4{\rm{n}}\beta \tau }$$

3 $$SNR\propto \sqrt{{E}_{{\mathrm{pulse}}}}\propto \sqrt{\tau }$$

The complex-domain matched filtering achieves pulse compression, breaking through the spatial resolution limit set by the original chirped signal duration and enabling high spatial resolution governed by the δ-pulse *FWHM*. Simultaneously, its excellent noise resistance concentrates signal energy, enhances the detectability of Raman backscatter, improves the system *SNR*, and extends the sensing range. Furthermore, the CEED method, through extracting the amplitude envelope from the complex-domain δ-pulse Raman scattering signal, effectively separates and suppresses phase fluctuation noise, significantly reducing the amplitude-phase coupling error caused by gain and phase fluctuations. This technique effectively suppresses phase noise while preserving the signal’s amplitude information.

Together, these features break the antagonistic limitations of pulse duration on traditional system performance and enable a balanced and synergistic enhancement of spatial resolution, sensing distance, and temperature accuracy of the ROTDR system.

### Properties of the complex-domain square-wave width-chirp pulse

To address the fundamental challenge that spontaneous Raman scattering obscures the frequency information of the optical source, we propose a complex-domain square-wave width-chirp scheme, in which the frequency information of the original chirp signal is modulated into the variation of the sub-pulse width of the width-chirp.

The expression for the traditional complex-domain sine chirp *s*(*t*) is given in Eq. (4).

4 $$s(t)=\exp \left[2\pi {\mathrm{i}}({f}_{0}t+\frac{1}{2}\beta {t}^{2})\right]$$

Where i is the imaginary unit, and *f*_0_ is the starting frequency of the chirp. The complex-domain square-wave width-chirp pulse signal, with its analytical expression given by:

5 $$x(t)={\mathrm{rect}}\left(\frac{t-2/\tau }{\tau }\right){\mathrm{sign}}\left({\mathrm{exp}}\left[\pi{\rm{i}}\left(\frac{t}{{W}_{0}}-\frac{{\beta }_{W}}{2{W}_{0}^{2}}{t}^{2}\right)\right]\right)$$

where rect(·/*τ*) is the rectangular window function of pulse duration *τ*, sign(·) denotes the sign function to generate the square wave. *W* is the sub-pulse width of the chirp pulse(*W*_0_ is the first sub-pulse width), *f* is the origin chirp signal’s instantaneous frequency. And *β*_W_ is the variation rate of the sub-pulse width. The relationship between *W*_0_, *W*, and *β*_W_ with respect to the original chirp parameters *f*_0_ and *β* is formulated in Eq. (6).

6 $${W}_{0}=\frac{1}{2{f}_{0}},W=\frac{1}{2f},{\beta }_{{\mathrm{W}}}=\frac{{\mathrm{d}}W}{{\mathrm{d}}t}=-\frac{\beta }{2{f}_{0}^{2}}$$

For the sake of clarity and consistency in the subsequent analysis, *f*_0_ and *β* are retained as the primary parameters for calculation and discussion throughout this work.

**Fig. 2:**
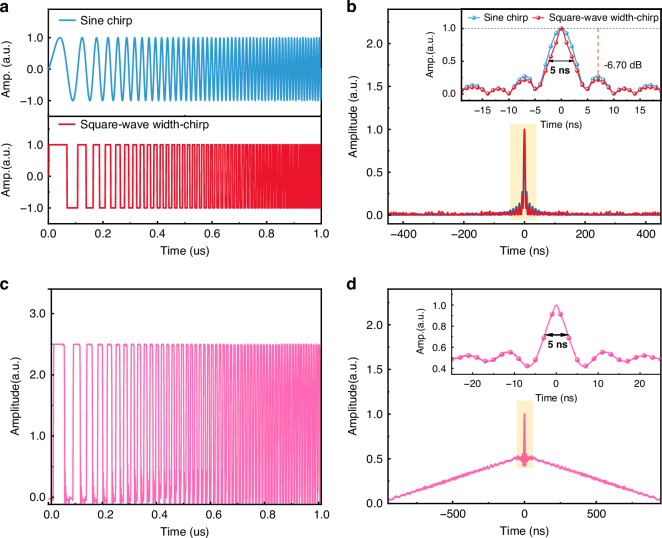
Properties of chirp signals. **a** Timing of sine chirp and square-wave width-chirp probe signals. **b** *FWHM* of the δ-pulse of the sine chirp and the square-wave width-chirp signal. **c** Timing of the square-wave width-chirp signal generated by PSG. **d** δ-pulses of PSG square-wave width-chirp signals. PSG pulse sequence generator

As shown in Fig. 2a, we compare complex-domain sine chirp and square-wave width-chirp signals. Their corresponding compressed δ-pulses, obtained via complex-domain matched filtering, are depicted in Fig. 2b. Benefiting from the approximately <1 ns rise time of the square wave and its high-order odd harmonic components, the effective bandwidth of the system is expanded, resulting in a *FWHM* of the compressed δ-pulse reduced to 5 ns and a sidelobe level suppressed to −6.70 dB. Theoretically, this enables higher spatial resolution.

In practical generation, the width-chirp waveform naturally forms a sub-pulse sequence that improves extinction ratio and system *SNR*. Such pulse groups can be efficiently generated and amplified using semiconductor optical amplifier modulation. The resulting optical pulse train offers a higher extinction ratio, thereby improving the overall system *SNR*. For these reasons, the square-wave width-chirp was ultimately selected as the probe signal in this study. Due to the unipolar nature of the chirp pulse used in the experiment, the resulting δ response contains a raised baseline. This baseline distortion can be effectively removed using a differential processing method^51^. Differential processing can reduce the impact of erbium-doped fiber amplifier transient effects, but it will introduce additional time costs and *SNR* decrease. In practice, a specific trade-off is needed.

### Spatial resolution

In the proposed CSWPC system, the spatial resolution is determined by the *FWHM* of the compressed δ-pulse. Under experimental conditions of *τ* = 1 μs and chirp rate *β* = 100 THz·s^−1^, Eq. (2) yields a calculated δ-pulse *FWHM* of 5 ns, corresponding to a spatial resolution of 0.5 m. This waveform was uploaded to the pulse sequence generator to generate the square-wave width-chirp pulse sequence (Fig. 2c). The compressed δ-pulse exhibits an *FWHM* consistently maintained at 5 ns, as shown in Fig. 2d, confirming the expected spatial resolution. Theoretical analysis indicates that, with a chirp pulse duration *τ* = 1 μs and a chirp rate of 100 THz·s^−1^, the achievable spatial resolution is 0.5 m.

To experimentally verify this, we inserted a 10 m section of fiber under test (FUT) at the 43 km position of the sensing fiber. The backscattered signal acquired by the avalanche photodiode (APD) was first transformed into the complex-domain to obtain the complex-domain Raman response. In experiments, a 10-order time-domain smoothing filter was applied to the raw backscattered signal in order to enhance data readability.

7 $$h(t)={x}^{\ast }(-t)$$

8 $$\hat{I}={\rm H}[I(t)],\,\hat{h}={\rm H}[h(t)]$$

**Fig. 3:**
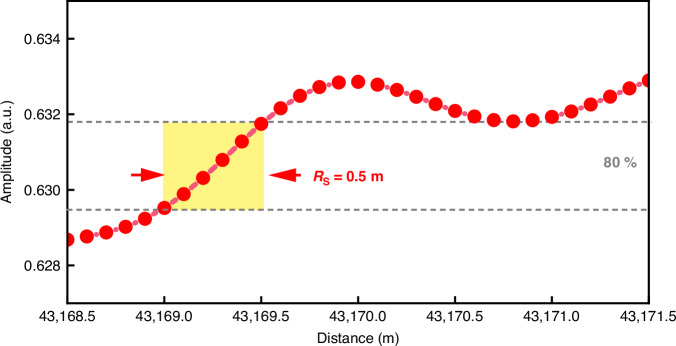
Spatial resolution *R*_s_ of the CSWPC scheme

A matched filter was then designed based on the reference signal acquired by the APD and constructed using Eq. (7). The complex-domain responses of the Raman scattering curve *I* and the matched filter h are obtained through the Hilbert transform H[·], as shown in Eq. (8). Complex-domain matched filtering was performed according to Eq. (9), ⊗ is a convolution operator. The FUT zone compressed δ-pulse Raman scatter trace signal is shown in Fig. 3.

9 $$\hat{M}=\hat{I}\otimes \hat{h}$$

By analyzing the 80% rising edge of the signal envelope corresponding to the inserted FUT, the spatial resolution was determined to be 0.5 m, consistent with the theoretical prediction. This result confirms that the resolution is governed by the *FWHM* of the compressed δ-pulse rather than the original pulse duration. Compared to conventional ROTDR systems using a 1 μs duration pulse, this method offers up to a 200-fold improvement in spatial resolution.

In traditional Raman distributed sensing systems, spatial resolution is directly determined by the pulse duration of the incident pulse due to the time-of-flight principle used in time-domain demodulation. In contrast, the proposed approach introduces square-wave width-chirp pulses and applies complex-domain matched filtering, enabling compressed δ-pulse responses without reducing the original pulse duration. Specifically, a matched filter is formed by taking the complex conjugate of the transmitted signal and convolving it with the received signal in the complex-domain. The resulting output exhibits a sharply narrowed main lobe—approaching a δ-pulse response—that corresponds to localized changes in Raman scattering intensity along the fiber.

This compression δ-pulse significantly improves the system’s ability to detect and localize short-length temperature events, thereby enabling spatial resolution performance that is independent of sensing range. The CSWPC scheme thus achieves ultra-high spatial resolution without sacrificing measurement distance or *SNR*, offering a compelling alternative to conventional time-domain systems.

### Sensing distance and temperature accuracy

**Fig. 4:**
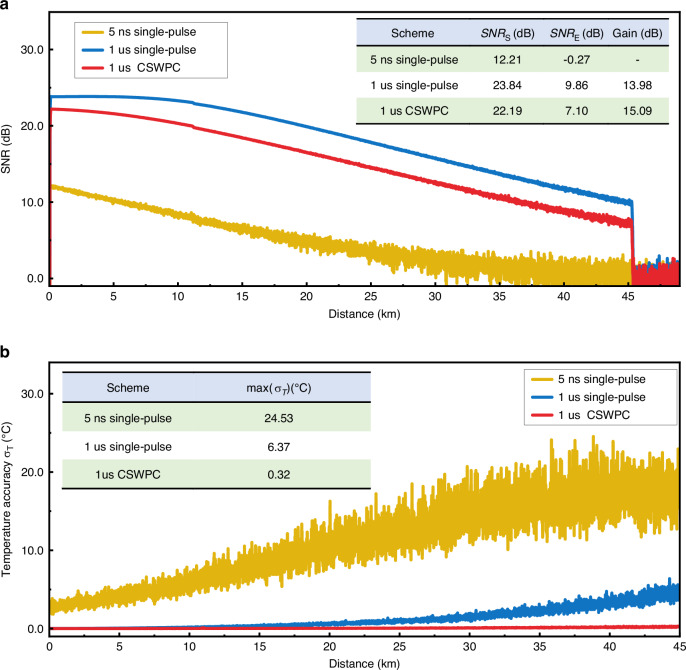
Effective sensing distance and temperature accuracy under different schemes. **a** *SNR* at effective sensing distances of different schemes. **b** Temperature accuracy of different schemes

Three sensing schemes were experimentally compared over a 45 km single-mode fiber link, including a 5 ns single-pulse demodulation scheme, a 1 µs single-pulse demodulation scheme, and the proposed 1 µs CSWPC scheme. The experimental results are presented in Fig. 4.

Figure 4a shows the evolution of the *SNR* with sensing distance for the three schemes, with the inset summarizing the *SNR* at the injection end (*SNR*_S_), and the *SNR* at 45 km (*SNR*_E_). For the 5 ns single-pulse scheme (yellow curve), the *SNR*_E_ becomes negative at 45 km, and the *SNR* gain is invalid, indicating that effective end-point signal detection cannot be achieved at this spatial resolution (0.5 m). When the pulse width is increased to 1 µs (blue curve), *SNR*_S_ and *SNR*_E_ improve to 23.84 dB and 9.86 dB, *SNR* gain is 13.98 dB, respectively, enabling a 45 km sensing range. However, according to the traditional ROTDR ranging principle, this scheme exhibits a spatial resolution of 100 m, insufficient to accurately resolve the 10 m FUT segments, highlighting the inherent trade-off in conventional single-pulse demodulation between long-range operation and high spatial resolution.

For the proposed 1 µs CSWPC scheme (red curve), *SNR*_S_, *SNR*_E,_ and *SNR* gain reach 22.19 dB, 7.10 dB, and 15.09 dB, respectively. Although *SNR*_S_ are slightly lower than those of the 1 µs single-pulse scheme, this reduction primarily arises from amplitude fluctuations in the square-wave chirp, which slightly reduce the effective energy flux. Nonetheless, subsequent complex-domain matched filtering and CEED processing compensate for this effect, ensuring that the difference does not materially impact the final temperature accuracy.

To quantitatively evaluate the temperature accuracy along the fiber, we demodulate the distributed temperature *T* based on Eq. (1), and compute, for each measurement point *l*, the standard deviation^21,25^ of temperature samples within an adjacent 10 m fiber segment, as defined in Eq. (10).

10 $${\sigma }_{T}=\sqrt{\frac{1}{N-1}{\sum ({T}_{{l}}-\bar{T})}^{2}}$$

Where *N* is the number of temperature samples within the 10 m segment and $\bar{T}$ is the mean temperature of that segment. The *σ*_T_ distributions for the three schemes under identical experimental conditions (25 °C) are shown in Fig. 4b. The yellow curve corresponds to the 5 ns scheme, the blue curve to the 1 µs scheme, and the red curve to the proposed CSWPC scheme. The inset lists the maximum for each case. For the 5 ns single-pulse scheme, *σ*_T_ increases significantly with distance, reaching approximately 24.53 °C at 45 km; this is consistent with the negative *SNR*_E_ observed in Fig. 4a, confirming that the 5 ns scheme cannot support effective sensing at 45 km. In the 1 µs single-pulse scheme, the higher pulse energy reduces *σ*_T_ to approximately 6.37 °C at 45 km, but a clear increase in *σ*_T_ with distance remains. With the 1 µs CSWPC scheme, complex-domain matched filtering combined with CEED maintains *σ*_T_ sub-degree Celsius across the entire 45 km fiber, with a *σ*_T_ of 0.32 °C at the end of the sensing range.

Traditional single-pulse demodulation schemes improve *SNR* by increasing the pulse width, but at the cost of degraded spatial resolution. In contrast, the CSWPC scheme redistributes the temporal energy to expand the time–bandwidth product and concentrates the energy into a narrow main-lobe through matched filtering, thereby achieving high spatial resolution and excellent temperature accuracy while keeping the total pulse energy constant. In the frequency domain, matched filtering enhances the signal’s main frequency band while suppressing out-of-band noise, whereas CEED further improves system robustness by decoupling amplitude and phase. The synergistic effect of these two techniques enables the CSWPC scheme to improve temperature accuracy by approximately 20-fold and 100-fold compared with the traditional 1 µs and 5 ns single-pulse demodulation schemes, respectively.

To quantitatively evaluate the synergistic enhancement effect of the complex-domain matched filtering and CEED two-stage denoising method on the far-end sensing performance, we selected five 10 m long sensing fiber segments (FUT-A to FUT-E) within the 41 km to 45 km region of the sensing fiber. Under steady-state temperature conditions, we collected and analyzed the following three types of temperature sample sequences: the original ROTDR demodulated temperature sequence, the temperature sequence after complex-domain matched filtering processing, and the final temperature sequence after further applying CEED denoising. The standard deviation of the temperature values in each sequence was used as the evaluation metric for temperature accuracy. The specific experimental results are shown in Table 1.

**Table 1:** Temperature accuracy of different processing stages

Processing stage	Sensing fiber segment				
	FUT-A	FUT-B	FUT-C	FUT-D	FUT-E
Original accuracy	5.96 °C	9.08 °C	6.58 °C	6.50 °C	7.18 °C
After matched filtering	0.15 °C	0.16 °C	0.16 °C	0.15 °C	0.17 °C
After CEED processing	0.11 °C	0.11 °C	0.12 °C	0.10 °C	0.12 °C

Analysis of the experimental data shows that the average temperature measurement standard deviation across the five fiber segments decreased significantly from the original 7.06 ± 1.21 °C (average of the five segments) to 0.16 ± 0.007 °C after complex-domain matched filtering, and was further optimized to 0.11 ± 0.008 °C after CEED. The corresponding temperature accuracy improvements were approximately 16.45 dB in the matched filtering stage and 1.62 dB in the CEED stage, resulting in a total improvement of 18.07 dB. In terms of contribution ratio, matched filtering accounts for about 91.0% of the total improvement, while CEED accounts for about 9.0%.

The experimental results demonstrate that complex-domain matched filtering is the primary mechanism for the step-like improvement in system *SNR* and temperature accuracy, effectively suppressing the dispersion of temperature samples. The CEED technique plays a further critical role at the far end of the fiber where the signal is weaker, effectively suppressing temperature drift. Working synergistically, they ultimately enable the system to maintain a temperature measurement accuracy of 0.11 °C even at 45 km, while significantly improving the spatial consistency along the temperature distribution.

Moreover, the matched filter in this method is identical to the probe signal itself. The total acquisition time is mainly determined by signal averaging: at a 2 kHz repetition rate, 2 × 10⁶ averages require ~16 min, while data-processing time is negligible due to optimized complex-domain matched filtering. Unlike pulse-coding methods, whose spatial resolution remains constrained by the code-bit pulse width and whose decoding requires computationally intensive matrix operations, our chirped-pulse-compression scheme determines resolution by the compressed δ-pulse width and enables fast, direct demodulation through complex-domain matched filtering. This achieves a fundamental breakthrough beyond the physical pulse duration limit while offering higher robustness, lower computational cost, and improved practical performance.

The proposed CSWPC scheme successfully achieves a synergistic balance of sensing distance, spatial resolution, and temperature accuracy in Raman distributed fiber optic sensing, offering a scalable and efficient solution for high-performance long-range temperature monitoring.

## Discussion

### Energy distribution mechanism of CSWPC

CSWPC enhances the time-bandwidth product (TBP) and converts it into matched-filter gain, enabling δ-pulse compression without sacrificing energy. The CSWPC scheme employs a hyperbolically modulated cluster of square-wave sub-pulses, which expands the effective Raman spectrum and increases the TBP by ~23 dB relative to a conventional 1 µs pulse. With the launched pulse energy kept constant, this enlarged TBP is translated into a processing gain through complex-domain matched filtering, yielding a compressed mainlobe that approaches a δ-pulse and providing ~22.19 dB *SNR* enhancement at the input end.

In contrast to traditional short-pulse strategies—where higher spatial resolution is achieved at the expense of pulse energy and long-range performance—CSWPC redistributes, rather than reduces, temporal energy. After matched-filter compression, the signal energy is concentrated into the δ-pulse mainlobe, enabling a 5 ns/0.5 m resolution while still maintaining 7.10 dB *SNR* at 45 km.

Because matched filtering suppresses noise variance in proportion to the TBP (≈1/200 of the 5 ns scheme baseline), CSWPC achieves a synergistic improvement in spatial resolution, sensing distance, and temperature accuracy within a unified framework.

### Performance under localized temperature variations

**Fig. 5:**
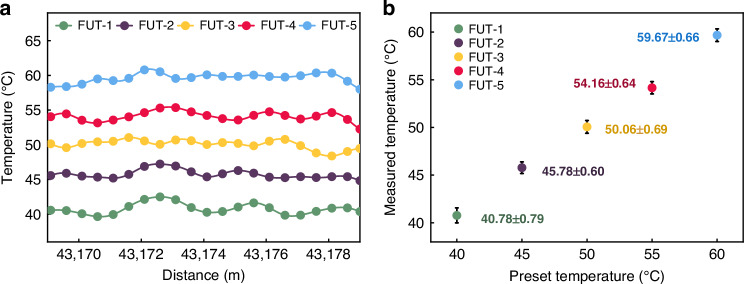
Distributed temperature demodulation results under FUTs at different surrounding temperatures. **a** Temperature demodulation curves. **b** Temperature accuracy and error bars

Under sharp local temperature transitions, the demodulation accuracy degrades slightly due to amplitude perturbations and chirp scaling-factor *A* uncertainty. To validate the CSWPC Raman distributed sensing system’s temperature sensing capability under varying thermal conditions, we deployed five FUT sections at the 43 km position of the sensing fiber—FUT-1 to FUT-5, each 10 m in length—set at temperatures of 40 °C, 45 °C, 50 °C, 55 °C, and 60 °C, respectively. The corresponding Raman backscattered signals were demodulated using Eq. (1). The final temperature demodulation curve of FUT is shown in Fig. 5.

As shown in Fig. 5a, the resulting temperature profiles clearly distinguish between the different temperatures, with accurate localization and amplitude differentiation. The extracted temperature average values and their associated measurement accuracies are summarized in Fig. 5b, indicating an average temperature accuracy of ±0.68 °C. The resolution of the reference thermometer used in the experiment was ±0.01 °C. Compared to the room-temperature condition reported earlier (±0.11 °C), a slight degradation in temperature accuracy is observed. This reduction is attributed to the localized amplitude perturbations in the Raman backscattered signal caused by sharp temperature transitions in the FUTs.

The CEED scheme performs optimally under constant or slowly varying amplitude conditions (such as uniform ambient temperature), while rapid thermal discontinuities introduce non-ideal distortions that reduce demodulation fidelity.

Furthermore, temperature accuracy is jointly determined by the uncertainties of Raman intensities, calibration temperature, and scaling coefficients. We can further explore temperature accuracy based on error propagation theory. By Eq. (1) and applying first-order error propagation theory, the error propagation relationship for the temperature measurement standard deviation *σ*_T_ is shown in Eq. (11).

11 $${\sigma }_{{T}}\propto \sqrt{\left(\frac{{\sigma }_{\mathop{M}\limits^{\frown {}}}^{2}}{\mathop{M}\limits^{\frown {}}}\right)+\left(\frac{{\sigma }_{{\mathop{M}\limits^{\frown {}}}_{0}}^{2}}{{\mathop{M}\limits^{\frown {}}}_{0}}\right)+{\left(\frac{{\sigma }_{A}}{A}\right)}^{2}+{\left(\frac{{\mathrm{h}}\varDelta \nu /{\mathrm{k}}}{{T}_{0}^{2}}\frac{\exp ({\mathrm{h}}\varDelta \nu /{\mathrm{k}}{T}_{0})}{\exp ({\mathrm{h}}\varDelta \nu /{\mathrm{k}}{T}_{0})-1}\right)}^{2}{\sigma }_{{T}_{0}}^{2}}$$

The calibration accuracy of the introduced amplitude scaling factor *A* in the model also directly affects the temperature demodulation results. The Eq. (11) indicates that the calibration deviation of *A* is the main cause of the systematic accuracy difference observed between the FUT and non-FUT zones in the experiments.

Temperature accuracy is used to describe the overall temperature uncertainty of a 10 m FUT. For the interior of the FUT, we further introduce the temperature peak-to-peak fluctuation^52^ *T*_p-p_ as an auxiliary evaluation metric. Its definition and calculation basis are given by Eq. (12):

12 $${T}_{{\mathrm{P}}{-}{\mathrm{P}}}=\,\max ({T}_{{l}\in \mathrm{FUT}})-\,\min ({T}_{{l}\in \mathrm{FUT}})$$

Based on Eq. (12), the calculated *T*_p-p_ for the five sections FUT-1 to FUT-5 in Fig. 5 are 2.86 °C, 2.39 °C, 2.66 °C, 3.17 °C, and 2.90 °C, respectively, with an average value of 2.80 °C. This metric intuitively reflects the temperature fluctuation amplitude within the FUT measurement zone.

### Influence of chirp parameters

**Fig. 6:**
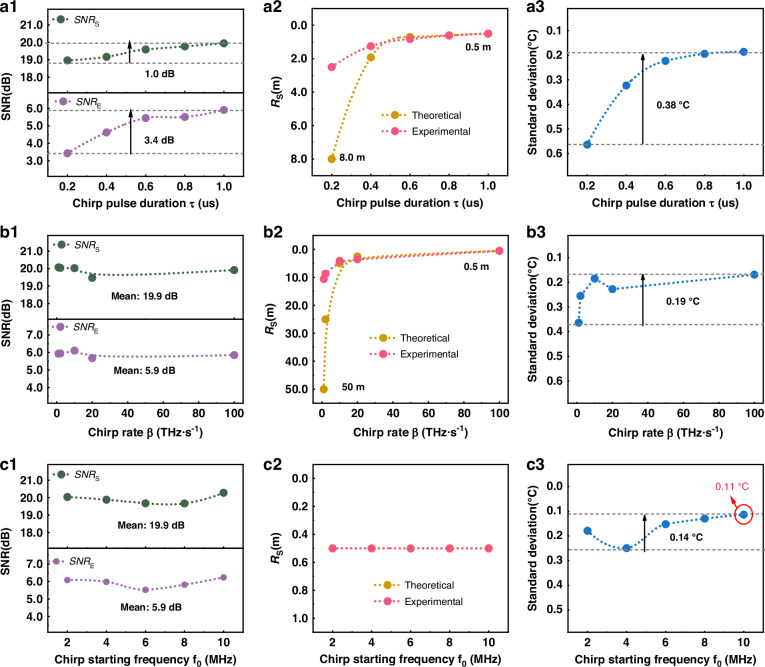
Sensing performance with different chirp parameters. **a** Performance of different chirp pulse durations. **a1** *SNR*, **a2** spatial resolution, and **a3** temperature accuracy. **b** Performance of different chirp rates **b1** *SNR*, **b2** spatial resolution, and **b3** temperature accuracy. **c** The performance of different starting frequencies. **c1** *SNR*, **c2** spatial resolution, and **c3** temperature accuracy

Pulse duration and chirp rate jointly govern resolution and *SNR*, with predictable trends aligned with Eq. (2) and Eq. (3). Equation (2) defines the theoretical relationship between spatial resolution and the parameters of the complex-domain square-wave width-chirp probe pulse. To validate this relation and further investigate the influence of pulse duration *τ*, chirp rate *β*, and starting frequency *f*₀ on overall system sensing performance, a series of experiments was conducted. The results of sensing performance with different chirp parameters are presented in Fig. 6. As shown in Fig. 6a, increasing the pulse duration leads to noticeable improvements in both *SNR*_S_ and *SNR*_E_. This is because a longer pulse carries more optical energy, thereby increasing the coupled power into the fiber and enhancing both the input and end-fiber *SNR*. Ultimately, this leads to improved sensing range. However, as observed in Fig. 6a1, the *SNR* improvement is not strictly linear. Due to limitations in device gain, the benefit from increasing pulse duration gradually saturates and eventually plateaus.

Figure 6a2 illustrates the effect of pulse duration *τ* on spatial resolution. According to Eq. (2), increasing *τ* improves spatial resolution from 8 m to 0.5 m theoretically, while the experimentally measured resolution improves from 2.4 m to 0.5 m. Notably, when the pulse duration falls below 200 ns, the experimental resolution outperforms the theoretical prediction. This is attributed to the system behaving effectively as a multi-subpulse system under short-pulse conditions, where spatial resolution is governed by the narrowest sub-pulse within the chirp waveform. Additionally, since *SNR* is positively correlated with temperature accuracy, increasing pulse duration to improve *SNR*_E_ also enhances temperature resolution. Specifically, the temperature accuracy at the fiber end improves from 0.57 °C to 0.19 °C, representing a 0.38 °C enhancement.

According to Eq. (2), the chirp rate *β* is also a critical determinant of spatial resolution. As *β* increases from 1 THz·s^−1^ to 100 THz·s^−1^, the theoretical resolution improves from 50 m to 0.5 m. When the chirp rate is low, the experimental resolution is again better than theoretical predictions because, under these conditions, the resolution is dominated by the narrowest local features of the chirp signal rather than its overall bandwidth. Both chirp rate *β* and starting frequency *f*₀ affect the temporal structure and spectral energy distribution of the probe, leading to nonlinear fluctuations in the effective coupled optical energy. These fluctuations cause oscillations in *SNR*_s_ and *SNR*_E_, which are illustrated in Fig. 6b3 and 6c3. While such variations do not compromise the system’s sensing range, they can cause slight instability in temperature accuracy. Nevertheless, an optimal temperature accuracy of 0.11 °C was achieved under specific chirp parameter configurations. Across the data, a strong positive correlation is observed between *SNR*_E_ and the temperature accuracy at the fiber end.

### Comparison with other advanced schemes

We focused on comparing key performance indicators like temperature accuracy and measurement time between our proposed scheme and other advanced technologies under the same or similar spatial resolution or sensing distance conditions. The comparison results are shown in Table 2.

**Table 2:** Sensing performance of various schemes

Scheme	Sensing distance	Spatial resolution	Temperature accuracy	Effective sensing points*	Time
Ultrashort pulse single-photon detection scheme^26^	0.08 km	0.37 m	0.9 °C	216	1.0 min
Pulse coding scheme^47^	39.0 km	1.0 m	3.9 °C	39,000	13.6 min
Few-mode fiber sensing scheme^44^	25.0 km	1.1 m	1.0 °C	25,000	1.5 min
Chaos correlation localization scheme^38^	10.0 km	0.3 m	2.0 °C	33,000	5.0 min
CSWPC Scheme	45.0 km	0.5 m	0.11 °C	90,000	16 min

*: The effective sensing points are defined as the ratio of the sensing distance to the spatial resolution, which can be used to evaluate both the sensing range and spatial resolution performance of a system simultaneously^21^

The ultrashort pulse single-photon detection scheme^26^ reported achieves a spatial resolution of 0.37 m and a temperature accuracy of 0.9 °C over a sensing distance of 0.08 km. While this category of methods can attain high spatial resolution, the sensing distance is generally limited due to the sharp decrease in pulse energy, resulting in a relatively small number of effective sensing points. Furthermore, when employing single-photon detection technology, the influence of polarization noise and interference noise cannot be neglected, which further constrains the system^26^. Additionally, the use of ultrashort pulse lasers and single-photon detectors increases the system hardware cost and complexity.

Represented by the Genetic-optimized coding (Go-code) scheme^47^, this approach achieves a spatial resolution of 1.0 m and a temperature accuracy of 3.9 °C over a sensing distance of 39.0 km, yielding approximately 39,000 effective sensing points. The non-periodic nature of this scheme eliminates the need for additional code switching and signal acquisition time, with a single acquisition time of 13.6 min. However, its high performance relies on the design of coding sequences based on distributed genetic algorithms and extensive experimental screening.

The few-mode fiber scheme used features a large effective mode area and low intermodal dispersion^44^. Under full few-mode fiber link conditions, this scheme achieves a temperature accuracy of 1.0 °C and a spatial resolution of 1.1 m over a distance of 25 km, resulting in approximately 25,000 effective sensing points. However, its system performance exhibits poor compatibility with commercial distributed temperature sensing systems; if a commercial single-mode DTS system is used, the temperature accuracy degrades significantly to 4.7 °C^44^ limiting its practical deployment flexibility.

The chaos correlation localization scheme reconstructs the Raman backscattering curve through time-domain differential processing and extracts the chaos amplitude carrying temperature information using chaos correlation compression. The chaotic asymmetric pulse paired scheme^38^ achieves a spatial resolution of 0.3 m, a temperature accuracy of 2.0 °C, and an acquisition time of approximately 5 min over a 10 km distance, with about 33,000 effective sensing points.

The CSWPC scheme, through the pulse compression mechanism, forms a δ-pulse response. Within this framework, the system’s spatial resolution is determined by the *FWHM* of the compressed δ pulse, rather than the original width of the transmitted pulse, thereby overcoming the direct constraint between pulse width and spatial resolution inherent in conventional methods. Furthermore, this scheme maximizes the output *SNR* by constructing a matched filter in the complex domain that is the conjugate time reverse of the Raman backscattering signal. Combined with the proposed CEED technique, it effectively separates and suppresses the phase noise in the Raman scattering signal, ultimately achieving a 15.09 dB gain in the system *SNR*.

The comparison results indicate that the proposed CSWPC scheme simultaneously achieves a high spatial resolution of 0.5 m and a temperature demodulation accuracy of 0.11 °C over a long sensing distance of 45 km. The number of effective sensing points reaches 90,000, significantly surpassing the comparative schemes mentioned above. Furthermore, this technology exhibits good compatibility with various optical fiber types, such as single-mode and few-mode fibers. Its measurement time is comparable to that of the Go-code scheme but does not require pre-designed coding sequences, demonstrating significant technical advantages and application potential in long-range, high-spatial-resolution ROTDR systems.

### Extensions and outlook

#### Limit of spatial resolution

The spatial resolution in the proposed scheme is determined by the *FWHM* of the compressed δ-pulse (Eq. (2)), breaking through the physical limitation imposed by the duration of the transmitted pulse width in traditional ROTDR systems. Therefore, in an ideal, homogeneous sensing fiber link, the spatial resolution itself does not degrade with increasing transmission distance. However, in practical systems, the decrease in *SNR* with increasing distance reduces the detectability of temperature change zones and increases the temperature measurement error *σ*_T_, which manifests in engineering terms as a gradual decline in event localization reliability and effective resolution capability.

At the engineering application level, the spatial resolution requirement can be moderately relaxed to trade off for higher *SNR* and temperature measurement accuracy. This can be achieved by introducing a spatial averaging length *L*_avg_. In this case, the effective spatial resolution *R*_s_^*^ of the system can be approximately expressed as the second norm combination of the original resolution and the averaging length (Eq. (13)). This relationship provides a theoretical basis for the flexible trade-off between spatial resolution and temperature accuracy in the system.

13 $${R}_{{\mathrm{s}}}^{\ast }=\sqrt{{R}_{{\mathrm{s}}}^{2}{+L}_{{\mathrm{avg}}}^{2}}$$

In experiments, besides the influence of Eqs. (2) and (13), the spatial resolution *R*_S_ is also constrained by the performance of the relevant hardware systems, thereby establishing distinct theoretical upper and practical lower limits. The theoretical upper limit of the *R*_S_ is primarily constrained by the digitization capability of the data acquisition system. The ADC used in this work has a sampling rate of 1 GSa·s^−1^, corresponding to a minimum resolvable time interval of 1 ns, which translates to an upper limit of 0.1 m for the *R*_S_ in optical fiber. The practical lower limit of the *R*_S_ is mainly determined by the analog bandwidth of the optoelectronic devices. In our system, the combined bandwidth of the modulation link, comprising the PSG, SOA, EDFA, and APD, is approximately 200 MHz. This limits the bandwidth of the chirped pulse that can be effectively generated and amplified, consequently defining the practical lower limit of the *R*_S_ to 0.5 m for our system.

It is important to note that the 0.5 m spatial resolution achieved in this experiment represents the current practical optimum under the present hardware configuration, fully validating the effectiveness of the CSWPC scheme. In the future, employing GHz-level optical modulation and amplification devices could push the gauge length towards the theoretical upper limit of 0.1 m imposed by the ADC. Furthermore, if combined with ADCs featuring higher sampling rates and optical modulators with a wider frequency range, it would be possible to surpass this upper limit and achieve even better spatial resolution performance.

#### Limit of temperature accuracy

Equation (11) indicates that the uncertainties of the various observed physical quantities involved in the temperature demodulation (such as Raman scattering intensity and calibration temperature) collectively influence the final temperature demodulation accuracy *σ*_T_, weighted by their corresponding sensitivity coefficients. Under the premise that the observed quantities are statistically independent and the system can be linearized around the calibration point, the contributions of the various error sources are additive and decoupled. Therefore, reducing the uncertainty of any observed quantity can proportionally decrease the overall temperature measurement error according to the magnitude of its sensitivity weight.

Furthermore, the proposed two-stage complex-domain denoising (complex match filtering and CEED) scheme improves the effective *SNR* without sacrificing spatial resolution, resulting in reduced fluctuation variance, higher temperature accuracy, and improved long-term system stability. By contrast, low-order (<10-order) time-domain smoothing provides only moderate variance reduction, while higher-order filtering leads to notable degradation in spatial resolution (e.g., broadened to 0.7 m with a 20-order filter). Wavelet-threshold denoising suffers from loss of high-frequency information; for example, using a 5-level dB3 wavelet causes the spatial resolution to deteriorate significantly to approximately 1.5 m. Should a light source with higher stability be employed, there remains potential for further improvement in the system’s temperature accuracy.

#### Limit of distance

In this study, the 45 km fiber was selected primarily based on experimental feasibility and reproducibility. This length corresponds to the maximum continuous single-span fiber spool available in our laboratory, which enables a stable link budget and system operation with a minimal number of fusion splices. Extending the sensing distance beyond 45 km would typically require concatenating multiple fiber spools and introducing additional connection points, thereby increasing insertion loss and uncertainty in the link loss. In practice, a longer fiber also often necessitates higher launch power, which may increase system instability and even trigger nonlinear effects; consequently, the optical architecture would need to be re-optimized.

Moreover, the 45 km fiber setting is close to the upper distance limit supported by the current repetition rate of our system (2 kHz, corresponding to a 500 μs sampling period). Longer sensing distances would require reducing the repetition rate to avoid temporal overlap. To maintain the sensing accuracy reported in this work, more cumulative averaging would typically be required as well, which would further prolong the acquisition time and reduce experimental stability and repeatability.

In addition, based on the measured *SNR*_S_(22.19 dB) and *SNR*_E_(7.10 dB) in Fig. 4a, we estimate the theoretical maximum sensing distance using a linear attenuation model. The calculation indicates an *SNR* decay rate of approximately 0.34 dB·km^−1^. Extrapolating this trend to an *SNR* of 0 dB yields a theoretical maximum sensing distance of 65 km. In future work, employing a higher-power pump laser, lower-noise amplifiers, or more sensitive detectors is expected to improve the link budget and thereby further extend the sensing range.

#### CSWPC scheme transfer

We hope that the proposed complex-domain square-wave width-chirp pulse probe, complex-domain matched filtering, and CEED method will offer valuable insight and practical benefit to other scattering–based fiber sensing systems, such as those utilizing Brillouin or Rayleigh scattering.

For example, the spatial resolution of traditional BOTDR/BOTDA systems is fundamentally constrained by the finite phonon lifetime, which limits the minimum usable pump-pulse duration. The proposed scheme has the potential to enhance the effective time–bandwidth product of Brillouin scattering systems, such that the sensing spatial resolution after matched filtering is primarily determined by the compressed pulse width. However, in Brillouin sensing systems, the accuracy of Brillouin gain spectrum (BGS) retrieval is intrinsically sensitive to the spectral properties of the pump pulse. Optical modulation and amplification stages typically introduce spectral broadening and frequency shifts, which may result in BGS broadening or distortion as well as reduced peak gain, ultimately degrading strain and temperature demodulation accuracy. Accordingly, translating the proposed complex-domain matched-filter pulse-compression framework to BOTDR/BOTDA requires dedicated optical designs or algorithmic compensation strategies to avoid, or compensate for, pump-spectrum-induced BGS deformation and associated measurement accuracy loss. Within these constraints, by combining long pump pulses with optical time–frequency multiplexing schemes—such as optical-chirped pulse chains^11^ or frequency-agile^53^—one may further develop a detection architecture capable of rapid BGS mapping, thereby jointly enabling high spatial resolution, high measurement speed, and high-precision distributed sensing.

In Rayleigh scattering systems, CSWPC can serve as a universal framework for signal enhancement and noise suppression. In a non-coherent OTDR^54^, pulse compression can significantly improve the detection capability for weak backscattered signals from the far end, effectively enhancing both the *SNR* and spatial resolution. In coherent φ-OTDR^55^, employing optimized chirp waveforms combined with complex-domain matched filtering can yield a narrower pulse response and lower sidelobe levels, which helps to improve the dynamic range of vibration phase demodulation and the system’s robustness. Further introduction of optical time-frequency chirp multiplexing and multi-carrier orthogonal designs could effectively suppress interference fading and polarization fluctuations, additionally enhancing system performance by increasing the equivalent pulse energy.

In summary, the CSWPC method proposed in this paper provides a universal pathway for performance enhancement in distributed optical fiber sensing systems based on various scattering mechanisms. In the future, through deep integration of this framework with different scattering mechanisms, it is anticipated that rapid, high-precision cooperative measurement of multiple physical parameters (such as temperature, strain, and vibration) could be achieved within a single sensing system, thereby advancing distributed optical fiber sensing towards multi-parameter and high-dimensional development.

## Materials and methods

### Temperature demodulation principle

At time *t* and temperature *T*, the Raman backscattered signal *I*(*t*) can be modeled as the convolution between the Raman characteristic equations *R*(*T,t*) and the probe signal *x*(*t*), as Eq. (14):

14 $$\begin{array}{l}I(t,T)=R(T,t)\otimes x(t)\\ {\rm{R}}(T,t)=\frac{{{\mathrm{K}}}_{{\mathrm{a}}}{\lambda }^{-4}}{\exp ({\mathrm{h}}\varDelta \nu /{{\mathrm{k}}}_{{\mathrm{B}}}T)-1}\cdot \exp [-({\alpha }_{0}+{\alpha }_{{\mathrm{s}}})\frac{{\mathrm{ct}}}{2{\mathrm{n}}}]\end{array}$$

Given that Raman backscattering yields a real-valued positive signal, the Hilbert transform is applied to construct its complex analytic signal, and the matched filtering is then performed in the complex-domain:

15 $$\hat{I}={\rm H}[I(t)]=\frac{1}{\pi }\int \frac{I(j)}{t-j}{\mathrm{d}}j$$

Where *j* is the integral variable. Then, the δ-pulse Raman scattering response $\mathop{{\rm{M}}}\limits^{\frown {}}$ is obtained through complex-domain matched filtering by Eq. (9). From the compressed signal, the temperature *T* at fiber location *L* can be retrieved using Eq. (1).

The physical constants and their reference values mentioned in this paper are defined in the following Table 3.

**Table 3:** Physical meaning and reference values of the constants mentioned

Constants	Physical meaning	Reference values
h	Planck constant	6.626 × 10^−34 ^J·s
k_B_	Boltzmann constant	1.38 × 10^−23 ^J·K^−1^
c	Light speed in a vacuum	3 × 10^8 ^m·s^−1^
n	Refractive index of SMF	1.5
K_a_	Raman anti-Stokes signal coefficient	3.066 × 10^−9^
Δν	Raman frequency shift	13.2 THz
*α*_0_+ *α*_s_	Transmission loss	1.18 dB·km^−1^
*λ*	Raman anti-Stokes signal wavelength	1450 nm

### Spectral characteristics of square-wave chirp

According to the Fourier series expansion theorem, the square wave can be expressed as a series of harmonic components dominated by odd orders, as Eq. (16). The superposition of *e*^i*kφ*^, and its amplitude coefficient decreases with the law of 1/|*k*| with the order. Therefore, square waves are inherently rich in higher-order odd harmonic components.

16 $$\begin{array}{cc}{\mathrm{sign}}[\cos \,\varphi ]=\sum {c}_{k}{e}^{{\rm{i}}{k\varphi}} & {c}_{k}=\left\{\begin{array}{ll}\frac{1}{\pi }\frac{1}{|k|}, & (k\,is\,odd)\\ 0, & (k\,is\,even)\end{array}\right.\end{array}$$

It is the introduction of these high-frequency harmonics that significantly expands the effective bandwidth of square waves, which in turn provides conditions for improving theoretical temporal resolution and compression performance. In OTDR systems, the spatial resolution is governed by the temporal resolution *Δt*, which is bounded by the Fourier limit Δ*t*·Δ*f* ≥ 1. Due to the higher-order harmonics in the square-wave, the spectral width Δ*f*_sq_ is broadened compared to sine chirps, approximated by Δ*f*_sq_ = Z·Δ*f*_sin_ with Z > 1. Therefore, theoretically:

17 $$\varDelta {t}_{{\mathrm{sq}}}\approx \frac{1}{\varDelta {f}_{{\mathrm{sq}}}} < \frac{1}{\varDelta {f}_{{\mathrm{sin}}}}\approx \varDelta {t}_{{\mathrm{sin}}}$$

As shown in Eq. (17), owing to its steep edges and the abundance of higher-order harmonic components, the square-wave width-chirp is expected to achieve a higher theoretical spatial resolution. Moreover, the existence of the square-wave width-chirp in the form of sub-pulse groups endows it with a higher extinction ratio, thereby leading to a superior system *SNR*.

### Theoretical formulation of spatial resolution and SNR

The sensing spatial resolution of a chirped pulse compression system is determined by the frequency sweep bandwidth Δ*F* of the chirped signal, as shown in Eq. (18)^49,55^.

18 $${R}_{s}=\frac{{\mathrm{c}}}{2{\mathrm{n}}\varDelta F}$$

Prior studies employ a time-symmetric chirp of duration τ*, whereas we design an up-chirped asymmetric sequence whose effective duration is *τ* = τ*/2. This changes the sweep-bandwidth relationship and leads to a modified spatial-resolution expression.

19 $${R}_{{s}}=\frac{{\mathrm{c}}}{2{\mathrm{n}}\varDelta F}=\frac{{\mathrm{c}}}{4{\mathrm{n}}\beta \tau }$$

The system *SNR* is defined as the logarithmic ratio of the effective signal power to the noise power, as given in Eq. (20). Furthermore, the *SNR* scales with the pulse duration *τ* according to Eq. (3).

20 $$SNR=10\,{\mathrm{lg}}\left(\frac{{P}_{\mathrm{signal}}}{{P}_{\mathrm{noise}}}\right)$$

### Experimental setup

**Fig. 7:**
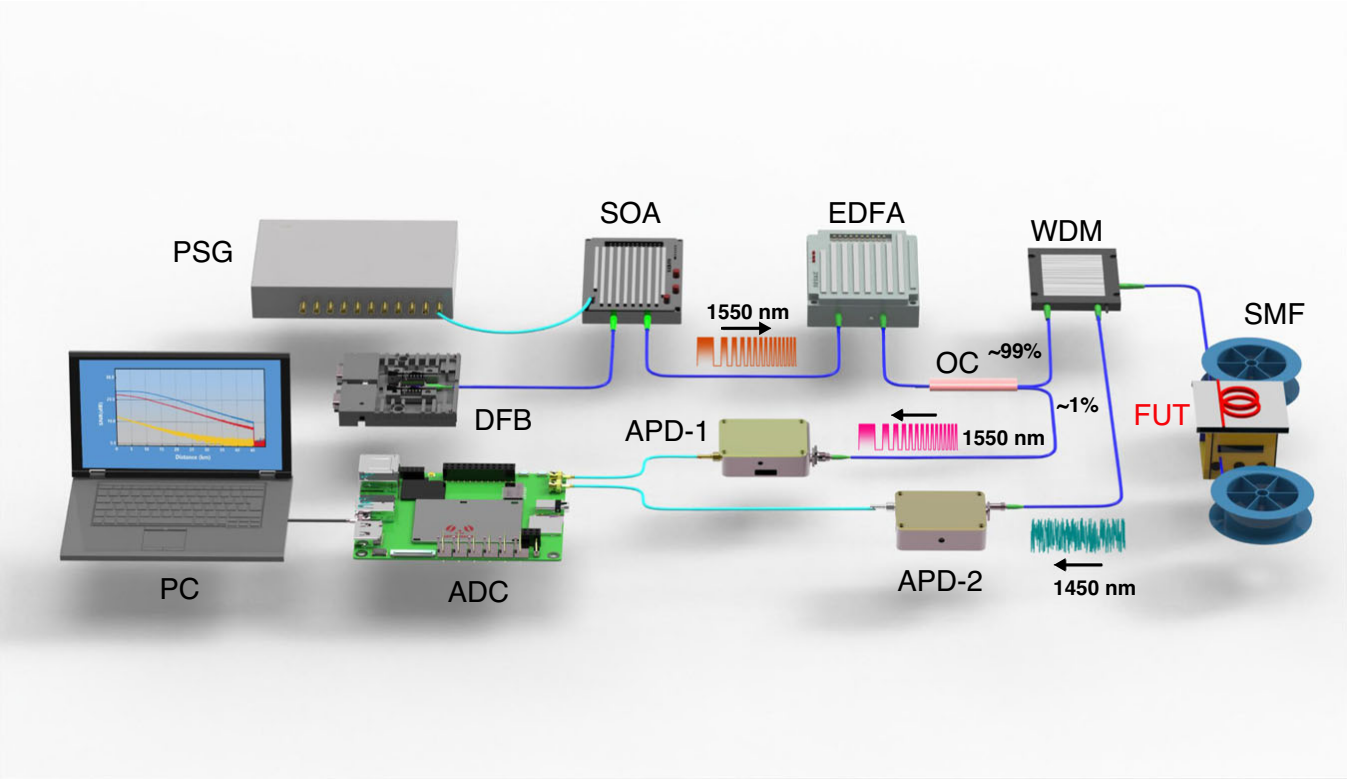
Experimental configuration of the CSWPC Raman distributed sensing. PSG pulse sequence generator, DFB distributed feedback laser, SOA semiconductor optical amplifier, EDFA erbium-doped fiber amplifier, OC optical coupler, WDM wavelength-division multiplexer, SMF single-mode fiber, FUT fiber under test, APD avalanche photodiode, ADC analog-to-digital converter, PC personal computer

A CSWPC Raman distributed sensing system was constructed, as shown in Fig. 7. A 1550 nm distributed feedback laser (DFB, HLT-DFB-M-1550-10-18-FA; center wavelength: 1546.92 nm; 3 dB-linewidth: 5 MHz; power fluctuation: <0.05 dB) serves as the light source. The square-wave width-chirp pulse probe sequence is first computed using Eq. (2) and downloaded into a pulse sequence generator (PSG, ASG8100; rise time: ≤1 ns; jitter: ≤35 ps), which drives a semiconductor optical amplifier (SOA, OAM-SOA-PL-15-15-S; operating wavelength: 1520–1570 nm) to generate the 1550 nm probe lightwave. The signal is then amplified by an erbium-doped fiber amplifier (EDFA, EDFA-C-PL-MB-100-S; operating wavelength: 1550 nm) and injected into a 1:99 optical coupler (OC). The 1% channel is detected by an avalanche photodiode (APD-1, KY-DTS-200M; 3 dB-bandwidth: 200 MHz) to obtain the reference signal and construct the matched filter. The 99% channel is launched into a 45 km single-mode fiber (SMF) via a wavelength-division multiplexer (WDM, WDM-1×3-1550; isolation: ≥60 dB). A temperature-controlled fiber under test (FUT) section is placed at the end of the fiber. The 1450 nm anti-Stokes Raman signal is filtered and detected by APD-2. Both signals are digitized using an analog-to-digital converter (ADC, DAQ2100; sampling rate: 1 GSa·s^−1^; jitter: ≤125 ps). Post-processing and demodulation are conducted on a personal computer (PC). The ADC sampling rate is 1 GSa·s^−1^, corresponding to a time step of Δ*t* = 1 ns. This translates to a distance step of 0.1 m per individual sample point. Adhering to the sampling quantization error, the spatial accuracy is stated as ±0.05 m.

We adopted a single-path anti-Stokes demodulation scheme because the anti-Stokes intensity provides higher temperature sensitivity, the scheme avoids dispersion-induced mismatch between Stokes/anti-Stokes channels, and it reduces hardware complexity and drift. This ensures improved spatial accuracy, *SNR*, and overall system robustness.

This scheme selected standard SMF as the sensing medium, primarily based on its excellent compatibility with front-end optical components (such as modulators, amplifiers, and WDM) and its well-established low-loss and controllable dispersion characteristics. To suppress the Fresnel reflection peak interference, an additional 100 m fiber pigtail was connected to the fiber end, shifting the reflection peak outside the sensing region and ensuring accurate end-fiber measurements. Applying index-matching gel at the fiber end is also an effective method for reducing Fresnel reflections by minimizing the refractive-index mismatch.

The chirp matching coefficient *A* is calibrated as follows: (a) Set the entire fiber to a uniform temperature *T*_0_, and acquire the baseline anti-Stokes signal *I*_0_. (b) Heat a defined segment of the fiber to a known temperature *T*, and record the new anti-Stokes signal *I*_T_. (c) Apply Eq. (1) to compute the coefficient *A*.

## Data Availability

Data underlying the results presented in this paper could be obtained from the authors upon reasonable request.
